# The Observation of Meiotic Union Behavior of Gametophytes Provides a New Basis for Ploidy of *Carassius auratus gibelio*

**DOI:** 10.3390/ani15020140

**Published:** 2025-01-08

**Authors:** Kexin Ma, Yueyao Yang, Yifan Li, Chuan Li, Taicheng Li, Haiyan Ma, Zibin Jiang, He Zhou, Wei Wang

**Affiliations:** Key Laboratory of Applied Biology and Aquaculture of Fish in Northern Liaoning Province, PRC, Dalian Ocean University, Dalian 116023, China; makexin20211029@163.com (K.M.); 13591122473@163.com (Y.Y.); lyf1767574356@163.com (Y.L.); pdfls828@163.com (C.L.); 18263078176@163.com (T.L.); mhy9012@163.com (H.M.); 17372083806@163.com (Z.J.)

**Keywords:** *Carassius auratus gibelio*, nucleolar organizer region, rediploidization, chromosome pairing

## Abstract

*Carassius auratus gibelio*, a significant polyploid fish in Chinese aquaculture, can reproduce through both unisexual gynogenesis and sexual reproduction. While *C. gibelio* is known to exhibit polyploidy throughout its lifecycle, whether the homologous pairing of chromosomes is associated with the mode of reproduction remains unclear. Our study determined its karyotype as 2n = 150, comprising 42 metacentric, 24 submetacentric, 6 subtelocentric, and 78 telocentric chromosomes. Using AgNORs, CMA_3_/DAPI double fluorescence staining, and FISH, we identified six nucleolar organizer regions in *C. gibelio* chromosomes. Female *C. gibelio* exhibited only univalent and bivalent configurations, while males showed a range of chromosome configurations from univalent to hexavalent. CMA_3_-positive sites and FISH signals in trivalents spermatocytes hint at homologous triploidization. The formation of two trivalents or two bivalents and two univalents from six NORs bearing chromosomes in male *C. gibelio* is considered evidence of being in the process of rediploidization. The presence of only univalents and bivalents in the oocytes of female *C. gibelio* indicates that their cytogenetics are more stable, suggesting that they have completed rediploidization. Asynchronous progression of polyploidization may lead to differences in reproductive patterns between male and female *C. gibelio*. Our results offer cytogenetic insights into the polyploidy and evolution of *C. gibelio*, providing foundational cytogenomics data. This study provides relevant references for revealing the distinct reproductive strategies of male and female *C. gibelio*: under stable conditions, gynogenesis enables rapid clonal reproduction and swift population growth, while sexual reproduction enhances the genetic diversity within the population.

## Simple Summary

Our study investigates the karyotype of a vital polyploid fish, *Carassius auratus gibelio*. We found 150 chromosomes with 6 nucleolar organizer regions, suggesting complex chromosomal interactions. Male and female *C. gibelio* show different chromosome pairings, indicating asynchronous evolution. The formation of two trivalents or two bivalents and two univalents from six NOR bearing chromosomes in male *C. gibelio* is considered evidence of being in the process of rediploidization. The presence of only univalents and bivalents in the oocytes of female *C. gibelio* indicates that their cytogenetics are more stable, suggesting that they have completed rediploidization. Our findings contribute to the understanding of the underlying mechanisms and evolutionary outcomes of this species’ genome. They provide relevant insights for further exploration of the reproductive modes of polyploid organisms and breeding strategies.

## 1. Introduction

As an important aquaculture fish, the genus Carassius exhibits different ploidy, including tetraploids and hexaploids [1,2]. Recent studies have identified the chromosomal composition of *C. gibelio* as AAABBB, with two sets of triploid chromosomes, which is hypothesized to have resulted from hybridization leading to the formation of polyploids [3]. Although *C. gibelio* cannot undergo the typical meiosis division showing a reduced fertility, the double-breeding pattern (unisexual gynogenesis and sexual reproduction) of *C. auratus* provides a new survival strategy for the species [4]. Earlier investigations proposed the possibility of bisexual reproduction in *C. gibelio*, where mature male individuals produce diploid sperm, potentially contributing to genetic diversity in the species [5]. Furthermore, it is hypothesized that *C. gibelio* may have undergone autotriploidization during evolution, allowing for the activation of eggs through sperm from closely related species in a process known as gynogenesis [6]. However, the cause of the difference in reproductive pattern between male and female is not clear. Whether polyploidy results in differences in reproductive patterns between males and females is not clear.

Polyploidization is one of the most important driving forces of species formation and evolution, including fish evolution, as doubling of the chromosome count occurs once or more in fish [7,8]. In particular, polyploidy can lead to the formation of polyvalents during the association phase of meiosis. By observing the formation of polyvalents during meiosis, we can reveal the process of biological evolution based on the origin and formation of polyploidy in *C. gibelio* [9], and this can inform us about its unique reproductive model through the behavior of chromosome recombination. The nucleolus organizer region (NOR), where the 18S + 28S rRNA genes reside on eukaryotic chromosomes, is an important site for rRNA production [10]. The number and location of NORs can also be used as an important indicator for the study of phylogenetic relationships among species [11]. The number of chromosomes ploidy level is usually the same as the number of NORs, and the ploidy of most species can be judged by the number of NORs. Therefore, observing the meiosis process during gametogenesis in *C. gibelio* and determining its chromosome homologous recombination patterns through the identification of nucleolar organizer regions (NORs) is of significant importance for studying its rare reproductive mode.

To elucidate the distinct polyploidization progress and patterns of homologous recombination in male and female *C. gibelio*, this study, based on determining chromosome numbers and karyotypes from kidney cell chromosome specimens, further ascertained their ploidy and the origin of polyploid chromosomes by counting the number of NORs during the metaphase of mitosis in kidney cells. The study also examined the process of homologous synapsis in the gametes of both sexes to determine differences in patterns of homologous recombination. This study provides an in-depth exploration of the ploidy and homology of the *C. gibelio* from a cytogenetic perspective, offering valuable insights into the biological characteristics of this species. Through meticulous chromosome analysis, we have revealed the composition and behavior of chromosomes in the gonads of different sexes of *C. gibelio*, which is crucial for uncovering its unique reproductive modes and evolutionary history. Our findings enhance the understanding of the genome structure and function of *C. gibelio* but also have significant implications for assessing its potential in aquaculture and optimizing breeding strategies.

## 2. Materials and Methods

### 2.1. Ethics Approval and Consent to Participate

The collection of, treatment, and experimental procedures on fish were conducted in accordance with the guidelines of the Dalian Ocean University (DLOU) and were approved by the Institutional Animal Care and Use Committee of DLOU (DLOU2024018).

### 2.2. Materials

The fish samples were collected from the aquaculture facility in Hulan, Heilongjiang Province, China and temporarily kept in the laboratory aquarium. Water conditions were consistently controlled throughout the trial. The temperature ranged from 22 °C to 24 °C and continuous aeration. We selected a total of four male and five female samples in good condition. The average body length and weight of the four males were 13.80 ± 2.71 cm and 90.20 ± 55.26 g, respectively. The average body length and weight of the five females were 24.6 ± 2.04 cm and 551 ± 48.16 g, respectively. We chose three males and two females randomly for karyotype analysis. All males (four in total) were used for observations of testicular meiotic division behavior, and the remaining three females were used for observations of ovarian oocyte meiotic division chromosomes.

### 2.3. Preparation of Chromosome Specimen of Nephrocyte

Three males and two females were chosen randomly for karyotype analysis. Since kidney cells have a high rate of mitotic activity, they are chosen as the material for determining the somatic chromosome number and karyotype [12]. Each fish was intraperitoneally injected with phytohemagglutinin (PHA injection doses: 6 µg g^−1^ body weight, Aladdin, Shanghai, China) to promote cell mitosis and followed by a second PHA treatment 18 h later to achieve a higher cell division index. Then, 4 h after the second PHA treatment, 0.1% colchicine (injection doses: 6 µg g^−1^ body weight, Beyotime, Shanghai, China) was intraperitoneally injected to keep most cells at mitotic metaphase [13]. After another 2 to 3 h, fish were anesthetized using 0.1% benzocaine (Kermel, Tianjin, China) for 10 min. Using scissors, the tail fin was cut off from the base, and the fish was bled for 10 min to ensure death. The kidney was taken out and placed in a small beaker of saline (7.5 g NaCl, 0.2 g KCl, 0.02 g Na_2_HCO_3_, and 0.2 g CaCl_2_ are dissolved in 1 L of distilled water, Aladdin). After preparing the sample into a cell suspension, take the supernatant in a centrifuge tube. Further mince the pellet, then add 1–2 mL of physiological saline and filter it through a 40 μm cell strainer (Biosharp, Hefei, China). Place the filtered cell suspension in the same centrifuge tube and centrifuge at 1600× *g* for 10 min. After removing the supernatant, treat with 0.075 mol L^−1^ KCl (Aladdin) for hypotonic treatment for 45 min, followed by centrifugation at 1600× *g* for 10 min. After removing the supernatant, the sample was fixed with Carnoy’s fixative (methanol 3: glacial acetic acid 1, Aladdin) and kept in a −20 °C freezer. Cell suspension was pipetted onto a glass slide that had been cleaned with chilled 95% alcohol and then air dried. The slides were stained with 10% Giemsa solution pH at 6.8 (Beyotime) for 1 h and then rinsed with distilled water.

### 2.4. Chromosome Counting and Karyotype Analysis

One hundred and fifty clear and well-distributed metaphase division phases were counted for five *C. gibelio* to ascertain the chromosome number, which, with good dispersion, a clear shape, moderate length, and no repeat metaphase division phase, were counted. The karyotype analysis was carried out in accordance with Li et al. [14].

### 2.5. The Preparation and Observation of the Meiotic Chromosomes in Egg Nuclei

Each female sample was injected into muscle with human chorionic gonadotropin (HCG) (injection dose: ♀20–25 IU·g^−1^) and heated to 25 °C [15]. After 4 h, fish were anesthetized using 0.1% benzocaine for 10 min. Using scissors, the tail fin was cut off from the base, and the fish was bled for 10 min to ensure death. The ovary was taken out from the body and placed in saline with estrogen (17α, 20β-dihydroxy-4-pregnen-3-one) and then cultured in the dark until the egg nucleus moved to the animal pole (every 10 min, 10 eggs were taken and placed in 4% glacial acetic acid for observation). After the egg nucleus reached the animal pole, the nuclei were isolated and the yolk was removed. The egg nucleus was placed in cold Carnoy’s fluid 3–4 h. Then the Carnoy’s fluid was changed and placed in a −20 °C freezer overnight. On the second day, the egg nucleus was placed on a slide for drying and then stained using DAPI (Roche, Shanghai, China) for 30 min and soaked in pure water for 30 min. We pressed the clean coverslip onto the slide. A Leica DM2000 fluorescence microscope (Leica Microsystems, Wetzlar, Germany) was used for observation, a Leica DF 450C CCD (Leica Microsystems, Wetzlar, Germany) was used for capturing images, and Photoshop was used for image processing.

### 2.6. Preparation and Observation of Chromosome Specimen of Meiosis in Testis

The gonad was taken out and placed in 0.075 mol L^−1^ KCl solution for 45 min. Then, the sample was placed in a fixative (methanol 9: glacial acetic acid 1) for 10 min, fixed with chilled Carnoy’s fixative, and kept in a −20 °C freezer overnight. A cell suspension was made from the testes of each male, and then one droplet was pipetted onto a glass slide that had been cleaned with chilled 75% alcohol. After drying at room temperature, the specimen was stained for 1 h with 10% Giemsa solution at pH 6.8.

### 2.7. Differential Staining

Differential staining was applied to the chromosome slides using the method of Li et al. [14].

The Ag-NORs staining procedure was referenced from the method of Howell et al. [16] and subsequently improved. Place the kidney cell chromosome slides of *C. gibelio* in a 65 °C constant temperature drying oven. Quickly mix 100 μL of 50% AgNO_3_ solution with 2% gelatin solution (Kermel), then evenly drop onto the chromosome slides and cover with coverslips. After treating in the dark at 65 °C for 1 to 2 min, when the chromosome slides turn a tea-brown color, remove the coverslips, rinse with 70 °C pure water, and air dry naturally.

The CMA_3_/DAPI double fluorescence staining method is referenced by Schweizer [17,18]. On the kidney cell chromosome slides of *C. gibelio*, evenly drop 150 μL of 0.5 mg/mL CMA_3_ (Sigma, Shanghai, China) treat in the dark for 45 min, then remove the coverslips and rinse in an MI buffer solution. Use a bulb pipette to dry the slides, place the slides in a staining bath with 0.1 mg/mL DAPI and stain for 15 min, then rinse again in the MI buffer solution. Dry the slides with a bulb pipette, and evenly drop 100 μL of mounting medium onto the slides, then store in the dark at 4 °C in a refrigerator.

### 2.8. Fluorescence In Situ Hybridization

Fluorescence In Situ Hybridization (FISH) was applied using human 5.8s + 28srDNA sequences as probes according to Fujiwara et al. [19]. For the preparation and detection of chromosome FISH probes in *C. gibelio*, we mixed 2 μL each of dATP, dGTP, dCTP, and 10 × buffer, 1 μL of 16-dUTP, 3.5 μL of enzyme solution (Roche), 1 μL of human 5.8s + 28s rDNA probe, and 6.5 μL of sterile water, then incubated at 15 °C for 2 h. Afterward, the mixture was treated at 65 °C for 10 min and centrifuged at room temperature. Sperm DNA and *E.coli* mixture was purified with 2.5 μL of 4 M ammonium acetate and 66 μL of 100% ethanol (Kermel), then hybridized with the probe at −80 °C for 20 min and at room temperature for 10 min. The purified probe was denatured at 75 °C for 10 min and placed on ice for 10 min. Chromosome slides were preheated at 65 °C for 2 h, then soaked in 50 mL of 20% Saline-Sodium Citrate Buffer (SSC) for 5 min. RNase was added and incubated at 37 °C for 30 min, followed by a rinse in 20% SSC. The slides were then treated with Carnoy’s fixative for 5 min and air-dried. For DNA denaturation, the RNase-treated chromosomes were incubated in 70% formamide at 70 °C for 2 min, followed by a freeze in 70% ethanol at −20 °C for 10 min, and finally in 100% ethanol at −20 °C for 5 min. For hybridization, a solution was prepared with Bovine Serum Albumin solution (BSA), SSC, sterile water, and dextran sulfate, mixed with a probe, then incubated at 37 °C for 30 min. The hybridization was carried out overnight at 37 °C. Post-hybridization washes were performed with wash solution A (50% formamide:10% SSC = 1:1) at 42 °C, followed by washes in wash solutions 10% SSC, 5% SSC, and 20% SSC for 20 min. Fluorescence detection involved mixing 1% BSA with the first antibody (FITC, Sangon Biotech, Shanghai, China) and the second antibody solution, applying to the slides, and incubating at 37 °C for 1 h. FITC washes were conducted in the dark on a shaker with wash solution B (20% SSC: Triton = 1000:1), and signal amplification was performed with 10% SSC at 37 °C for 1 h.

### 2.9. Chromosomes and Hybridization Signals Observation

A Leica DM2000 fluorescence microscope (Leica Microsystems, Germany) was used to observe chromosomes and hybridization signals, a Leica DF 450C CCD (Leica Microsystems, Germany) was used for capturing images, and Photoshop (Adobe, 2021) was used for image processing. CMA_3_ and FISH signal points are excited using blue light (λ = 440~480 nm), while chromosomes stained with DAPI are excited using ultraviolet light (λ = 200~380 nm).

## 3. Results

### 3.1. Chromosome Number and Karyotype of C. gibelio Kidney Cells

**Figure 1:**
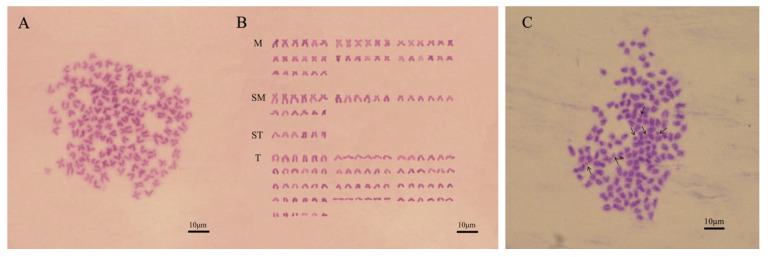
The metaphase and karyotype of *C. gibelio* renal cell chromosomes; (**A**) Staining of *C. gibelio* (**B**) karyotype of *C. gibelio*; M, metacentric chromosome; SM, submetacentric chromosome; ST, sub telocentric chromosomes; T, telocentric chromosomes. (**C**) Microchromosomes in *C. gibelio*. The black arrow indicates microchromosomes.

In this experiment, from the 90 cells that were counted, 5 cells that showed clear division were selected for the karyotype analysis. The results indicated that the. *C. gibelio* with 2n = 150 chromosomes were categorized into 42 metacentric (m), 24 submetacentric (sm), 6 subtelocentric (st), and 78 telocentric (t) chromosomes; no morphologically distinct sex chromosomes were identified (Figure 1A,B). In addition to the 150 basic chromosomes, we also observed 0–6 microchromosomes with small sizes (Figure 1C). The microchromosomes in *C. gibelio* are smaller than the normal chromosomes. Overall, the karyotype formula for *C. gibelio* is 2n = 150 = 42m + 24sm + 6st + 78t, with the presence of additional microchromosomes.

### 3.2. Analysis of Chromosome Banding in C. gibelio Kidney Cells

**Figure 2:**
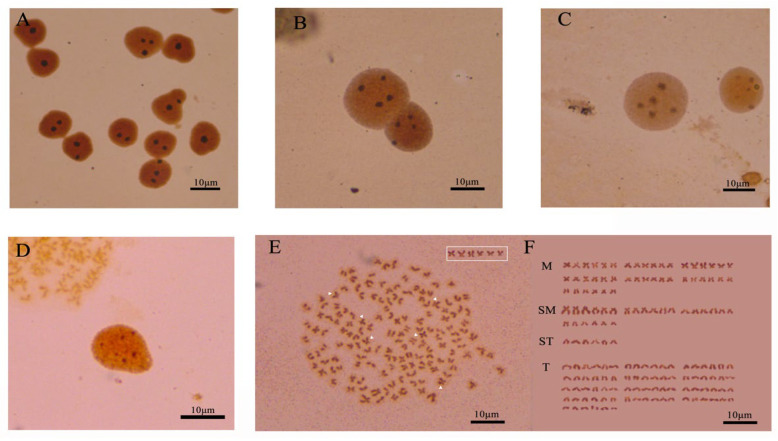
Ag-NORs stained of *C. gibelio* (**A**–**E**); (**A**) interphase nuclei with 1–3 silver-stained (**B**) interphase nuclei with 4 silver-stained (**C**) interphase nuclei with 5 silver-stained (**D**) interphase nuclei with 6 silver-stained (**E**) Ag-NORs stained metaphase chromosomes of *C. gibelio* somatic cells (**F**) karyotype of *C. gibelio* somatic cells. The white box and arrow indicate chromosomes with silver-stained spots.

The silver-stained cells of *C. gibelio* showed polymorphism, which was mainly reflected in the different numbers of Ag-NORs in the interphase nuclei and metaphase phases of the chromosomes. In this study, three hundred silver-stained interphase nuclei of *C. gibelio* kidney cells were observed and analyzed. The number of Ag-NORs was 1–6 (Figure 2A–D). One hundred well-dispersed metaphase phases with clear silver staining sites were observed and analyzed. The number of Ag-NORs was 1–6, and up to 6 Ag-NORs could be observed. The highest and most common number of staining sites was six, at 29% (Table 1). The short-arm end-region of the chromosome was located in the third group of middle centromeric chromosomes, which are the terminal Ag-NORs (Figure 2E,F).

**Figure 3:**
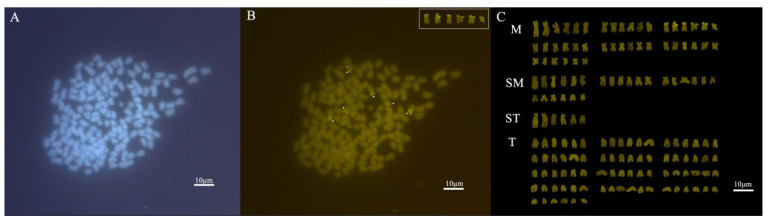
(**A**) DAPI staining of *C. gibelio* somatic cell, (**B**) CMA_3_ staining of *C. gibelio* somatic cell, (**C**) karyotype of *C. gibelio* somatic cells. The white box and arrow indicate chromosomes with CMA_3_ fluorescence signal points.

After CMA_3_/DAPI double fluorescence staining treatment of the kidney cell chromosome specimens of *C. gibelio*, well-dispersed metaphase cells were observed under the microscope. By observing and counting 30 cells with clear signals and a chromosome count of 150 in the metaphase of mitosis, it was found that the number of signal spots ranged from 1 to 6. Under ultraviolet light excitation (λ = 200~380 nm), up to six faintly stained DAPI fluorescence bands could be observed in the metaphase of chromosomes; under blue light excitation (λ = 440~480 nm), six bright and clear CMA_3_ fluorescence signal points could be observed in the chromosome division phase. Further karyotype analysis revealed that the six CMA_3_-positive sites were located at the end of the short arm of the central metacentric chromosomes of the third group (Figure 3).

**Figure 4:**
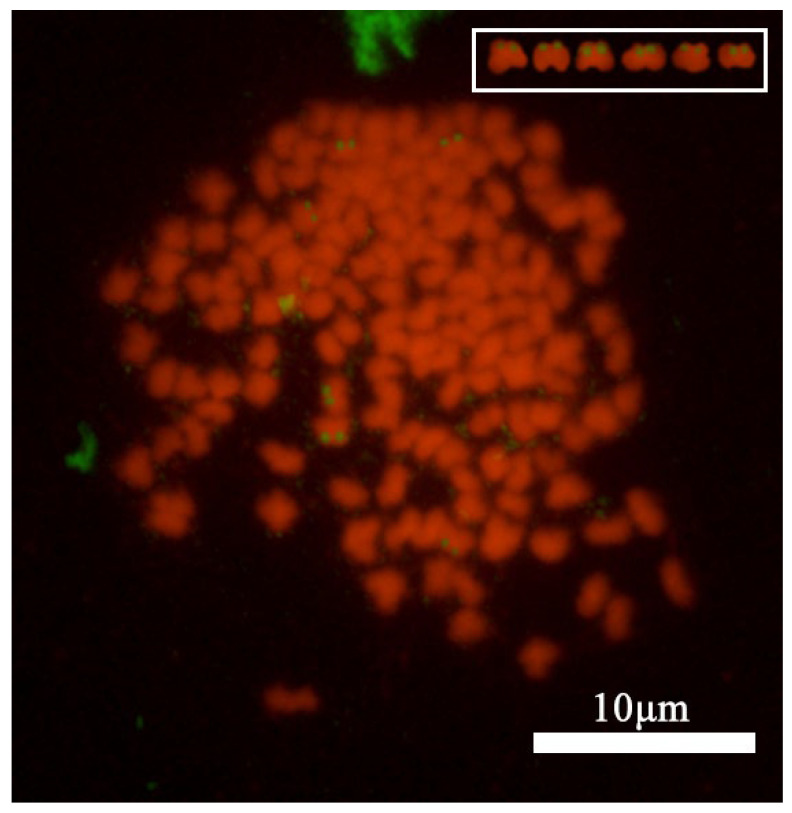
Metaphase spreads of *C. gibelio* with FISH signals obtained using 18S rDNA probe.

Using 5.8s + 28srDNA of human as a probe, we studied the metaphase chromosomes of the kidney cells of *C. gibelio*. The results showed that six hybridization signals occurred in *C. gibelio* (Figure 4). According to the karyotype analysis, hybridization signals were located at the end of the short arm of metacentric chromosomes, the same location that was observed with Ag-NORs and CMA_3_/DAPI double fluorescent staining.

Overall, *C. gibelio* has six NORs, which are all located at the end of the short arm of metacentric chromosomes.

### 3.3. The Observation of Chromosome Behavior for Oocyte Meiosis

**Figure 5:**
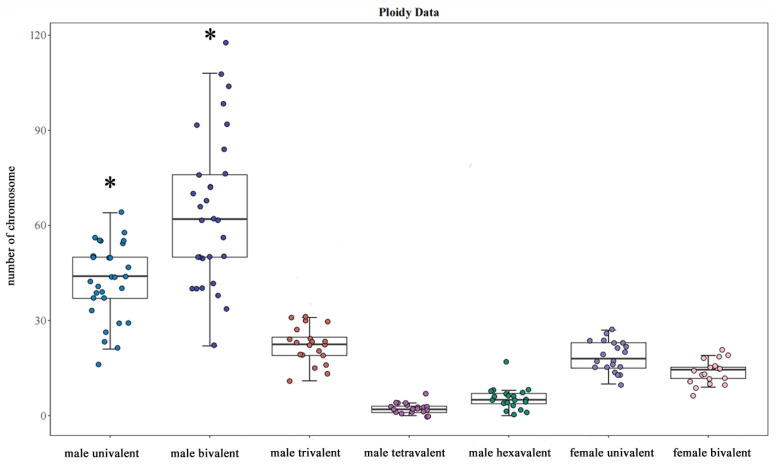
The quantity of univalent, bivalent, and polyvalent in egg nucleus and spermatocyte of *C. gibelio*. A plus (*) indicates significant differences.

**Figure 6:**
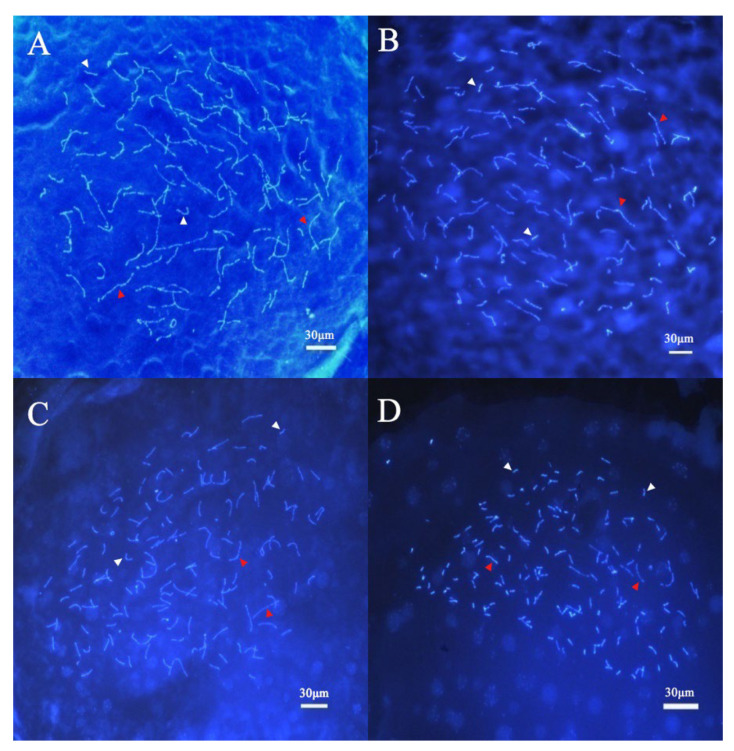
DAPI-stained meiotic configurations in oocyte germinal vesicles comprising from *C. gibelio*. (**A**) 50I + 50II, (**B**) 68I + 41II, (**C**) 72I + 39II, (**D**) 92I + 29II; white arrows, univalents; red arrows, bivalents. I, univalents; II, bivalents.

We observed the chromosomes of thirty oocytes from three female samples. The results showed that the oocyte chromosomes of female *C. gibelio* were composed of univalents and bivalents. An average of 62 univalents and 44 bivalents were counted from 30 oocytes of female *C. gibelio* (Figure 5). There were many kinds of chromosome pairing patterns in the egg cells of *C. gibelio* (Table 2), including 50I (univalents) + 50II (bivalents), 68I + 41II, 72I + 39II, and 92I + 29II (Figure 6).

Overall, the chromosome number of female *C. gibelio* oocytes conforms to (22–118) I + (16–64) II = 150, which is consistent with the chromosome number of kidney cells.

### 3.4. The Observation of Chromosome Behavior for Permatocyte Meiosis

**Figure 7:**
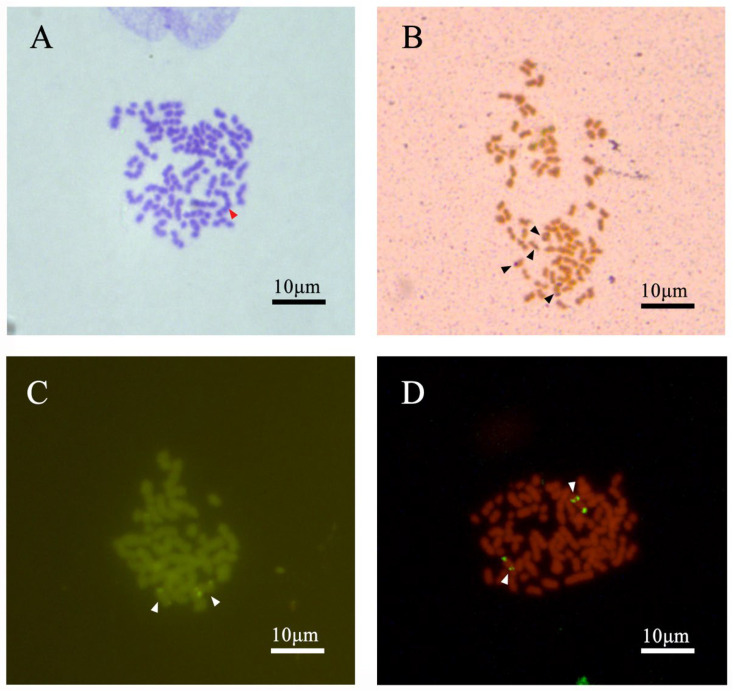
The chromosome division phase in different staining states of *C. gibelio* spermatocytes; (**A**) chromosome division phase by Giemsa; red arrows, hexavalent (**B**) chromosome division phase by Ag-NORs, The black arrows indicate the multivalents of the site (**C**) chromosome division phase by CMA_3_, The white arrows indicate the multivalents of the site (**D**) chromosome division phase by FISH, The white arrows indicate the multivalents of the site.

A total of twenty sperm cell chromosome configurations in four male *C. gibelio* were observed. During the first meiotic anaphase, the chromosomes in the germ cell were composed of univalent (I), bivalent (II), trivalent (III), quadrivalent (IV), and hexavalent (VI) compositions. (Figure 7) The univalent was short and rod-shaped, the bivalent was chain-shaped, and the polyvalent was long and chain-shaped. An average of 14–15 univalents, 22–23 bivalents, 18 trivalents, 5 quadrivalents, and 2 hexavalents were observed in the spermatocytes of male *C. gibelio* (Figure 5). A total of 20 different pairings (Table 3) were observed. The order of the organisms based on their chromosome count is as follows: (6–21) I + (11–31) II + (10–27) III + (0–17) IV + (0–7) VI = 150. The number of univalents and bivalents in males is significantly higher than that of other multivalents.

Most of the three staining methods identified six signal sites. After silver staining, the spermatocyte chromosomes showed six silver staining points, distributed as univalent and bivalent compositions (Figure 7B). The CMA_3_ staining and the signal points passing through the FISH showed that six sites were only distributed on trivalents (Figure 7C,D).

## 4. Discussion

Characteristic chromosomal variation is considered a key cause of population differentiation and a reflection of the evolution of fish’s adaptation to the wild environment [20]. This is evidenced by the significant differences in the karyotype composition of *Pelteobagrus fulvidraco* populations in Heilongjiang and Hubei under different habitat conditions [21,22]. In this study, we discovered that *C. gibelio* from our sample exhibits a chromosome count of 150 but displays distinct karyotypes, similar to *C. auratus* from Pengze City, Jiangxi Province, China [23] and *C. carp* in Caohai from Guizhou Province, China [24]. These results suggest that the variation in chromosome characteristics in fish is partly caused by different long-term geographical isolation and genetic factors. In addition, 156 chromosome counts have been reported by Shen et al. [25], which is different from the 150 in our study. The Qihe Crucian Carp chromosome counts were 156 [26] and 162 [27], which may indicate a potential for differentiation in the same habitat. The presence of microchromosomes may be one of the reasons for the differences in chromosome counts [28]. In addition to the 150 basic chromosomes, 0–6 microchromosomes were also found in this study. Therefore, it can be inferred that the karyotype evolution of *C. gibelio* has undergone the process of aneuploidy based on the large variation in chromosome count and the phenomenon of a higher number of microchromosomes.

In this study, silver staining, CMA_3_/DAPI double fluorescence staining, and FISH all showed 1–6 NORs in metaphase located in the terminal region of the short arms of the centromere chromosome, suggesting that there are several homologous sequences in the six middle centromeric chromosomes, and that *C. gibelio* has six sets of chromosomes. The number of NORs in the chromosomes of *C. auratus* from different regions is different. The fish from Dian Lake, Yunnan province, have four and six NORs [29], while the fish from Japan have two NORs [30]. The differences in the number of NORs may be related to the differences in the structure, number, and function of the chromosomes [31]. These differences may affect the ability of fish to grow, reproduce, and adapt to their environment [32].

To study the process of chromosome homology, we observed the chromosome configuration in the sperm cells and egg nuclei of *C. gibelio*. In male *C. gibelio*, there are hexavalent as well as univalent, bivalent, trivalent, and quadrivalent configurations. It is possible that their allotetraploid ancestors experienced subsequent autotriploidy [7], resulting in six highly similar homologous chromosomes. The diversification of these homologous chromosomes may be due to twinning differences between the triploids. Structural heterogeneity within the homologous chromosome is considered evidence of subsequent doubling. After rediploidization, modern fish exhibit more stable cytogenetic capabilities due to the decrease in the number of polyvalent chromosomes and the increase in the number of bivalent chromosomes, leading to more equal distribution of gametes and better genetic stability [15]. The presence of only univalents and bivalents in the oocytes of female *C. gibelio* indicates that their cytogenetics are more stable, suggesting that they have completed rediploidization. According to the results of karyotype and NORs staining, a hexavalent configuration was expected to be found in the nuclear chromosomes of the female *C. gibelio*, but, in fact, only bivalents and univalents were found. Bivalents were equally distributed, while univalents were randomly distributed. The germ cell behavior of male and female *C. gibelio* is different. In our previous studies on the male and female gonadal cells of *Misgurnus anguillicaudatus*, we found no relationship between sex and chromosome pairing [15]. These findings indicate the polyploid origin of the *C. gibelio* and subsequent whole-genome doubling. The meiotic chromosomal configurations of males and females are different; however, it seems that the latter process is still ongoing in males, as evidenced by the presence of hexavalent and polyvalent chromosomes, as well as diploids. This may be related to the unique reproduction of *C. gibelio*. The polyvalent structure indicated that the gonadal cells of these polyploid animals were more diversified than those of diploid animals. Our results found that for both sexes, the same *C. gibelio* within the same population has undergone a significantly asynchronous evolutionary process, preliminarily proving that due to the rich genetic diversity within the species, the fish have undergone adaptive differentiation early in their life.

We also used silver staining, CMA_3_/DAPI double fluorescence staining, and FISH to localize the spermatocyte chromosomes of *C. gibelio*. Interestingly, although the spermatocytes showed six signal spots in all three methods, the locations of the signal spots were different: Ag stains only appeared on the bivalent and monovalent of spermatocyte cells. This indicates that the male *C. gibelio* is in the process of rediploidization. However, the six CMA_3_-positive NOR sites and FISH fluorescence signal spots were only found on two trivalent chromosomes, which was consistent with the result that *C. gibelio* had two sets of triploid chromosomes. In addition, we found that five FISH signals were strong and one was weak. The existence of strength differences in rDNA signals has been widely demonstrated, including in *C. gibelio*. This phenomenon is most likely due to the different degrees of chromosome condensation or rDNA sequence duplication [33]. We hypothesized that because each nucleolar organizer region may undergo an unequal exchange of sister monomers at the time of division, the amount of replication of rDNA at the time of replication may result in differences in signal strength [34]. The discordance of signal point positions may be due to different chromatin structures and nucleolar organizations, which may affect the binding efficiency of probes to target sequences, thus affecting signal generation and presentation [34]. Furthermore, no FISH signals or CMA_3_-positive sites were found on the rDNA sites and NORs of the hexavalents, but FISH signals and CMA_3_-positive sites were found in both trivalents. This phenomenon illustrates the existence of structural heterogeneity in this trivalent, which is found in both the rDNA sites and NORs in tetraploid *M. anguillicaudatus* somatic cells [15]. In addition, in some polyploid amphibians, the existence of different pairing patterns in the homologous chromosome is due to the structural heterogeneity of the chromosomes [35,36]. The formation of two trivalents or two bivalents and two univalents from six NOR bearing chromosomes in male *C. gibelio* is considered evidence of being in the process of rediploidization. The presence of only univalents and bivalents in the oocytes of female *C. gibelio* indicates that their cytogenetics are more stable, suggesting that they have completed rediploidization [15]. The variation in signal point display locations, as observed in *C. gibelio*, indeed reaffirms the presence of NORs polymorphism. This polymorphism is a well-documented phenomenon in the field of cytogenetics, with studies highlighting the extensive polymorphism of rDNA clusters within and between subspecies. Understanding the extent of NORs polymorphism can provide insights into chromosomal dynamics and evolutionary processes. Further studies are necessary to resolve the mode of NORs evolution and shed light on the genetic and evolutionary relationships within and among species [37].

## 5. Conclusions

This study demonstrated that *C. gibelio* has 150 chromosomes and that its karyotype is 2n = 150 = 42m + 24sm + 6st + 78t. Six nucleolar regions were found in the metaphase division phase of the chromosome, which proved that *C. gibelio* is hexaploid. Hexavalent, bivalent, trivalent, and tetravalent germ cells were found in male *C. gibelio*. In female *C. gibelio* germ cells, only univalent and bivalent cells were found. These results suggest that the evolutionary processes of male and female *C. gibelio* are not synchronous: males may undergo rediploidization, while females may have completed this process. Furthermore, the presence of CMA_3_-positive sites and fluorescence in situ hybridization signals in the trivalents in spermatocytes further suggested that *C. gibelio* may have undergone homologous triploidization. The difference in signal point display location once again proved the existence of a nucleolar organizer region polymorphism. Additional studies are needed to confirm this.

## Figures and Tables

**Table 1:** Summary of Ag-NORs staining sites from *C. gibelio* at each metaphase and nucleolus in nucleus at interphase.

The Number of Ag-NORs Site	Number of Metaphases	The Number of Interphase Nuclei in a Single Cell	Number of Nucleus
1	2	1	57
2	14	2	82
3	13	3	71
4	26	4	48
5	16	5	28
6	29	6	14

**Table 2:** Number of chromosome specimens.

Conformation	Number	Frequency
118I + 16II	1	3.3%
108I + 21II	1	3.3%
104I + 23II	1	3.3%
98I + 26II	1	3.3%
92I + 29II	2	6.7%
84I + 33II	1	3.3%
76I + 37II	2	6.7%
72I + 39II	2	6.7%
70I + 40II	1	3.3%
68I + 41II	1	3.3%
66I + 42II	1	3.3%
62I + 44II	3	10.0%
56I + 47II	1	3.3%
50I + 50II	5	16.7%
42I + 54II	1	3.3%
40I + 55II	3	10.0%
38I + 56II	1	3.3%
34I + 58II	1	3.3%
22I + 64II	1	3.3%

I, univalents; II, bivalents.

**Table 3:** Number of bivalents and quadrivalents in the testicular cells from male *C. gibelio*.

Conformation	Number	Frequency
6I + 11II + 10III + 17IV + 4VI	1	5%
9I + 24II + 17III + 6IV + 3VI	1	5%
10I + 24II + 20III + 8IV	1	5%
10I + 30II + 14III + 8IV + 1VI	1	5%
11I + 30II + 17III + 4IV + 2VI	1	5%
12I + 23II + 16III + 5IV + 4VI	1	5%
12I + 13II + 26III + 5IV + 3VI	1	5%
13I + 19II + 27III + 3VI	1	5%
13I + 27II + 15III + 8IV + 1VI	1	5%
14I + 19II + 22III + 5IV + 2VI	1	5%
15I + 22II + 15III + 7IV + 3VI	1	5%
15I + 23II + 19III + 5IV + 2VI	1	5%
15I + 31II + 13III + 7IV + 1VI	1	5%
15I + 15II + 13III + 6IV + 7VI	1	5%
16I + 16II + 21III + 4IV + 4VI	1	5%
16I + 19II + 24III + 6IV	1	5%
17I + 31II + 15III + 3IV + 2VI	1	5%
19I + 20II + 23III + 1IV + 3VI	1	5%
19I + 31II + 15III + 3IV + 2VI	1	5%
21I + 22II + 23III + 1IV + 2VI	1	5%

I, univalents; II, bivalents; III, trivalent; IV, quadrivalent; VI, hexavalent.

## Data Availability

The original contributions presented in this study are included in the article. Further inquiries can be directed to the corresponding author(s).
